# Building Skills in Infection Prevention Through Simulation: Insights from Nursing Students in Brazil and Peru

**DOI:** 10.3390/nursrep16010014

**Published:** 2026-01-06

**Authors:** Luciene Muniz Braga, Pedro Paulo do Prado-Junior, Andréia Guerra Siman, Talita Prado Simão Miranda, Mara Rúbia Maciel Cardoso do Prado, Luana Vieira Toledo, Rodrigo Siqueira-Batista, Andréia Patrícia Gomes, Yanet Castro Vargas, Luis Alberto Chihuantito-Abal, Edo Gallegos Aparicio, Miluska Frisancho Camero, Sdenka Caballero Aparicio, José Efraín Larrea Campos, Kelly Myriam Jiménez de Aliaga, Zoila Isabel Cárdenas Tirado, Rosario del Socorro Avellaneda Yajahuanca, Isaías Wilmer Dueñas Sayaverde, Nely Esperanza Mundaca Constantino, María Itila Díaz Coronel, Antonio Sánchez Delgado, Edwin Barboza Estela, Maria Antonieta Rubio Tyrrell, Anibal Obtlitas Gonzáles, Raquel Guzmán Ordaz, Eva María Picado Valverde, Juan Antonio Juanes Méndez, María José Fermoso Palmero, Belén García Sánchez, Amaia Yurrebaso-Macho, Elisabete Pimenta Araújo Paz, Margareth Cristina de Almeida Gomes, Sabrina da Costa Machado Duarte, Francimar Tinoco de Oliveira, Priscila Brigolini Porfirio Ferreira, Anabela Salgueiro-Oliveira, João Graveto, Filipe Paiva-Santos, Maria da Conceição Bento, Manuel Chaves, Paulo Santos-Costa, Pedro Parreira, Teresa Neves

**Affiliations:** 1 Departmento de Medicina e Enfermagem, Universidade Federal de Viçosa, Viçosa 36570-900, Brazil; pedro.prado@ufv.br (P.P.d.P.-J.); ago@ufv.br (A.G.S.); talita.prado@ufv.br (T.P.S.M.); mara.prado@ufv.br (M.R.M.C.d.P.); luana.toledo@ufv.br (L.V.T.); rsbatista@ufv.br (R.S.-B.); 2 Núcleo de Bioética e Ética Aplicada, Centro de Ciências da Saúde, Universidade Federal do Rio de Janeiro, Rio de Janeiro 22290-902, Brazil; andreiapgomes@gmail.com; 3 Facultad de Ciencias de la Salud, Universidad Andina Del Cusco, Cusco 08006, Peru; ycastro@uandina.edu.pe (Y.C.V.); lchihuantito@uandina.edu.pe (L.A.C.-A.); egallegosa@utea.edu.pe (E.G.A.); mfrisancho@uandina.edu.pe (M.F.C.); scaballeroa@utea.edu.pe (S.C.A.); jlarrea@uandina.edu.pe (J.E.L.C.); 4 Facultad de Ciencias de la Salud—Escuela Profesional de Enfermería, Universidad Nacional Autónoma de Chota, Chota 06120, Peru; kjimeneza@unach.edu.pe (K.M.J.d.A.); zcardenas@unach.edu.pe (Z.I.C.T.); rsavellaneday@unach.edu.pe (R.d.S.A.Y.); iduenias@unach.edu.pe (I.W.D.S.); nemundacac@unach.edu.pe (N.E.M.C.); midiaz@unach.edu.pe (M.I.D.C.); asanchezd@unach.edu.pe (A.S.D.); ebarbozae@unach.edu.pe (E.B.E.); tyrrell2004@hotmail.com (M.A.R.T.); aoblitas@unach.edu.pe (A.O.G.); 5 Escuela de Enfermería de Zamora, The University of Salamanca, 37008 Salamanca, Spain; r.guzman@usal.es (R.G.O.); evapicado@usal.es (E.M.P.V.); jajm@usal.es (J.A.J.M.); euemjfer@usal.es (M.J.F.P.); belengs@usal.es (B.G.S.); amaiay@usal.es (A.Y.-M.); 6 Escola de Enfermagem Anna Nery, Universidade Fedral do Rio de Janeiro, Rio de Janeiro 21211-110, Brazil; elisabetepaz@eean.ufrj.br (E.P.A.P.); margarethgomes@eean.ufrj.br (M.C.d.A.G.); sabrinaduarte@eean.ufrj.br (S.d.C.M.D.); francimartinoco@eean.ufrj.br (F.T.d.O.); priscilabrigolini@eean.ufrj.br (P.B.P.F.); 7 Escola Superior de Enfermagem da Universidade de Coimbra (ESEUC), University of Coimbra, 3004-011 Coimbra, Portugal; anabela@esenfc.pt (A.S.-O.); jgraveto@esenfc.pt (J.G.); filipesantos@esenfc.pt (F.P.-S.); cbento@esenfc.pt (M.d.C.B.); mchaves@esenfc.pt (M.C.); paulocosta@esenfc.pt (P.S.-C.); parreira@esenfc.pt (P.P.); teresa_neves@esenfc.pt (T.N.)

**Keywords:** nursing, education, simulation training, cross infection, learning

## Abstract

**Background/Objectives**: Healthcare-associated infections (HAIs) require specific skills in nursing education, yet their curricular integration often remains fragmented, limiting the consolidation of knowledge and safe clinical practice. This study aimed to explore the perceptions of nursing students from Brazil and Peru regarding the use of clinical simulation as a strategy to develop skills in HAIs prevention and control. **Methods**: A qualitative approach was employed, involving 12 focus groups (n = 297 students) across four universities. The discussions were conducted following simulation activities based on standardized scenarios structured into four phases: pre-reading, briefing, execution, and debriefing. Data were collected using a semi-structured interview guide flowed by content analysis, through which saturation was achieved. The study adhered to COREQ guidelines. **Results**: Three main themes emerged: (i) clinical simulation as a student-centered teaching–learning strategy, where pre-reading and briefing materials enhanced students’ confidence and clarity in performing tasks, with checklists suggested to avoid omissions; (ii) simulation as a facilitator of autonomy and safety in HAI prevention, offering a protected environment for making mistakes and learning, with formative feedback during debriefing increasing risk awareness, although debriefing time was noted as an area for improvement; and (iii) meaningful learning and integration with traditional education, as students reported increased engagement, better knowledge retention, and greater perceived transfer of skills to real clinical settings. **Conclusions**: Clinical simulation demonstrated strong potential to support the development of HAI prevention skills in undergraduate nursing students. Longitudinal implementation with standardized scenarios and further evaluation of educational effectiveness and debriefing strategies is recommended.

## 1. Introduction

Healthcare-associated infections (HAIs) remain a persistent challenge to patient safety and care quality, requiring nursing students to develop strong skills in infection prevention and control during their undergraduate education. Despite institutional efforts, the literature highlights the fragmentation and inconsistency of HAIs-related content within nursing curricula, along with only moderate levels of knowledge and adherence to essential practices such as hand hygiene, an area emphasized by the international patient safety agenda [1,2,3,4]. This educational gap hinders the acquisition and integration of the knowledge, skills, and attitudes necessary for safe clinical practice, reinforcing the need to improve and standardize undergraduate nursing curricula [5,6].

To meet the growing demands of healthcare systems, nursing education must support the development of skills necessary for comprehensive, humanized, and solution-oriented care [7]. As frontline providers, nurses are in a strategic position to detect complications early and implement interventions that reduce adverse events [2]. Therefore, undergraduate programs should integrate theoretical and practical content with clear learning objectives that promote clinical decision-making from the start of professional training [8]. Among the pedagogical strategies that support these goals, clinical simulation stands out.

Clinical simulation is widely recognized as an active learning methodology that bridges theory and practice, enhances student engagement, and promotes meaningful learning. It allows students to develop clinical reasoning, technical skills, and self-efficacy in a controlled, risk-free environment, especially when supported by structured briefing and debriefing [9,10,11].

Organizations such as the World Health Organization (WHO) and initiatives like Quality and Safety Education for Nurses (QSEN) recommend integrating simulation into health education due to its demonstrated benefits for learning and transfer to clinical practice [3,9]. International studies, including those conducted in Asia and Latin America, report gains in knowledge and the development of infection prevention skills for both students and faculty, reinforcing the value of clinical simulation in teaching HAI-related topics [12]. However, most existing studies focus on measurable outcomes such as knowledge acquisition or technical performance, with limited exploration of how students perceive simulation-based learning in the context of HAI prevention. Comparative research that examines these perceptions across different national and institutional settings, particularly in Latin America, remains scarce. Gaining insight into students’ experiences is essential to inform curriculum development, ensure cultural relevance, and support the broader integration of simulation-based strategies in nursing education.

In this context, the HAInnovPrev project, a collaborative initiative between institutions in Brazil, Peru, Spain, and Portugal, aims to strengthen HAI prevention training in Latin America. One of its key innovations is the incorporation of clinical simulation into undergraduate nursing education. This study aimed to explore the perceptions of nursing students in Brazil and Peru regarding simulation-based teaching as a strategy to develop skills in HAIs prevention and control, providing evidence to support curriculum development and the expanded use of active learning methodologies.

## 2. Materials and Methods

### 2.1. Study Design and Theoretical Framework

This qualitative study used focus group interviews and conducted simulation activities to explore nursing students’ perceptions of clinical simulation scenarios as a strategy for developing skills in HAIs prevention. The descriptive–exploratory design was based on simulation as an active teaching–learning methodology and was guided by Kolb’s Experiential Learning Theory [13]. According to this framework, the simulation activities are organized according to the experiential cycle, in which the students: (1) undergo experiences similar to the care context during the simulated scenarios; (2) make a reflective observation in the structured debriefing; (3) reconstruct abstract concepts by relating their performance to evidence-based protocols for the prevention of HAIs; and (4) plan active experimentation for future clinical practice. This cyclical process favors the development of clinical reasoning, autonomy and essential skills for patient safety [13].

### 2.2. Participants and Recruitment

Purposive sampling was used to select students who met the profile of the simulated scenario. In total, 663 students were invited individually and/or by e-mail; 480 participated in the simulation activities, but 183 refused to participate in the final stage of the study (focus groups), resulting in a final sample of 297 students. It was not possible to identify the reasons for non-participation.

Undergraduate nursing students enrolled at two universities in Brazil and two in Peru were eligible to participate. Students engaged in short-term international academic mobility and those not fluent in Portuguese or Spanish were excluded.

The recruitment of participants happened in a structured and standardized way, conducted in each institution by professors who were members of the research team who were not involved in the academic evaluation of students, in order to minimize possible biases of coercion or hierarchical influence: (1) Face-to-face approach: Students were approached during face-to-face curricular activities, in the classroom and in practical activities in simulation laboratories, where they received oral explanations about the objectives, procedures and voluntary nature of the research; (2) Approach through institutional e-mail systems: e-mails were sent to students, containing detailed information about the study and request for confirmation of interest. The free and informed consent form was given to the students on the day of the simulated scenario activity, before starting the focus group.

### 2.3. Context, Scenario and Research Team

The study took place as part of the HAInnovPrev Erasmus+ project (Project number: 101083115—ERASMUS-EDU-2022-CBHE, Capacity Building in Higher Education), involving four institutions: the Universidade Federal de Viçosa (UFV) and the Federal University of Rio de Janeiro (UFRJ) in Brazil, and the Universidad Andina del Cusco (UAC) and Universidad Nacional Autónoma de Chota (UNACH) in Peru.

There is a growing need for nursing Higher Education Institutions (HEIs) to incorporate, in a structured way, teaching content and strategies aimed at the prevention and control of healthcare-associated infections (HAIs). Recommendations from international organizations, such as PAHO and WHO, and national guidelines indicate that weaknesses in initial training compromise the ability of future professionals to work with safety and quality in care.

The process of inclusion of the institutions occurred through previous contact with professors from several institutions in Latin America, and those who showed interest in improving pedagogical practices within the scope of HAI were selected, only these four universities agreed to participate in the study. In addition, a situational diagnosis was carried out within the scope of the HAInnovPrev project, showing that in the four participating HEIs, the teaching of HAI had low visibility in the curricula, content distributed in a fragmented manner and absence of clear learning objectives in the teaching plans. In addition, reports from students, professors and nurses of the health services that receive these students pointed out that many enter the clinical field with insufficient knowledge to apply basic measures for the prevention and control of HAIs, reinforcing the need for pedagogical review and standardization.

Therefore, four universities with diverse institutional characteristics and inserted in different sociocultural contexts were selected, allowing a multicentric analysis. In Brazil, the Universidade Federal de Viçosa (UFV), a public university with a 15-year nursing course and in the process of pedagogical consolidation, and the Anna Nery School of Nursing of UFRJ (EEAN/UFRJ), a historic institution with 100 years of experience in nursing education, therefore a national reference, but which also presented weaknesses in the curricular integration of HAI contents. In Peru, the Universidad Andina del Cusco (UAC), a private institution of regional relevance, developing teaching with active methodologies and expanding in the internationalization of teaching, and the Universidad Nacional Autónoma de Chota (UNACH), a public university, with a nursing course created 15 years ago and has a strategic role in the training of professionals for remote areas. In all institutions, the diagnosis indicated the need to strengthen the teaching of HAI in an articulated, explicit and continuous way throughout the training.

By integrating institutions with different profiles, the study sought to strengthen the quality of higher education in nursing in both countries, promote essential competencies for safe practices, and expand the alignment of curricula with the contemporary demands of health systems.

### 2.4. Data Collection

Data collection for the focus groups was conducted immediately after the simulation debriefings using a semi-structured interview guide with the following questions: (i) How did participating in this clinical simulation scenario improve your knowledge about HAIs? (ii) How did the pre-reading materials provided before the simulation help guide your learning about HAIs? (iii) How did the debriefing enhance your skills or understanding of HAIs? (iv) In what ways did participating in this simulation add value to your HAI-related skills compared to traditional learning methods?

The interviews were conducted in Portuguese in Brazil and in Spanish in Peru. To ensure linguist and conceptual equivalence across countries, the template with the interview questions was translated by one of the researchers from each country and reviewed by a second researcher, the project coordinator, fluent in the respective languages. The questions were further adapted to the linguistic and cultural context of each country to guarantee clarity and relevance for participants.

The simulation scenario scripts were developed by professors from each university within the HAInnovPrev consortium, based on a standardized template validated by the InovSafeCare project [12]. The template included: (i) clinical and non-clinical learning objectives; (ii) theoretical foundations with pre-reading materials; (iii) scenario preparation including material, human resources, and environment; (iv) scenario development detailing clinical cases, roles of actors and students, expected progression; and (v) a performance checklist to enable interinstitutional replication; and questions for the debriefing [14]. These scripts were reviewed and refined in meetings by five professors from the Nursing School of the University of Coimbra in Portugal, to ensure consistency and credibility. A pilot implementation of the first simulation scenario was conducted in May 2024 with students from one partner institution, followed by a transnational alignment meeting to discuss methodological consistency and preliminary impressions. No changes were required in the interview guide or data collection procedure as a result of this pilot. UFRJ did not participate in the pilot because the institution was still awaiting ethics approval at that time.

The simulation sessions followed a structured, sequential format. First, the pre-simulation phase involved distribution of theoretical materials 24 to 48 h in advance. Next, the briefing phase provided orientation including learning objectives and scenario presentation within a psychologically safe environment, using low-fidelity mannequins or trained actors or teachers. This was followed by the execution phase, during which students engaged in the scenario, applying their knowledge and skills in a controlled setting. Finally, the debriefing phase consisted of a facilitated critical reflection that highlighted successes and areas for improvement [15,16].

All sessions were audio-recorded and stored on a secure institutional server with restricted access. Recordings were transcribed immediately after the focus groups and anonymized using alphanumeric codes. After quality checks, the audio files were deleted. Transcripts were stored in a password-protected folder. Member checking, or returning transcripts to participants for validation, was not conducted. However, any ambiguities were clarified during the debriefing sessions.

Data collection took place between April 2024 and October 2024, except for the UFRJ, where data collection began later, in July 2024, due to delays in obtaining ethics committee approval. All activities were conducted in simulation laboratories. No individuals other than the participants and the researcher-facilitator were present during the focus groups. Each institution developed three HAI-focused scenario roadmaps and implemented a common scenario created by one of the partners. A description of the scenarios is provided in Table 1.

Although focus groups traditionally include between 6 and 12 participants, in multicenter studies there is methodological flexibility, considering a large sample of students, in which thematic saturation occurs between groups and not necessarily within each individual group.

The number of participants was defined based on the methodological indicators foreseen in the HAInnovPrev project (Erasmus+ CBHE), which established the minimum participation of 75 students exposed to four simulated scenarios per institution. Therefore, each university conducted four focus groups, corresponding to the four simulated scenarios implemented locally. In addition, participation in the scenarios occurred according to the students’ interest, with no previous limitation of number by the investigators, which resulted in natural variation in the number of participants, reflecting the voluntary nature of participation and ensuring that all scenarios were discussed, allowing the achievement of thematic saturation at the intergroup level.

In all, 16 focus groups were conducted (four per institution), totaling 297 students (Table 2).

### 2.5. Data Analysis

The testimonies were analyzed using content analysis [17], by three researchers in four meetings, and the thematic categories were validated by all researchers involved in data collecting at a transnational meeting of the HAInnovPrev project in December 2024. Data collection concluded upon clear evidence of data saturation, in accordance with the methodological framework employed in this study [17], meaning that no new insights emerged and no additional focus groups were required once thematic redundancy was reached. The stages of organization, coding, and categorization followed established procedures described in the literature, with themes inductively derived from the data. Analytical categories were supported by representative excerpts from students’ testimonies, ensuring coherence between the data corpus and the resulting interpretation. This transparent analytic trail ensured a clear link between the raw data and the resulting categories. For each simulated scenario, a single focus group session was sufficient, as data saturation were achieved [18].

Although the focus groups were conducted in the four participating universities, the thematic analysis was initially conducted separately in each institution, which allowed the identification of preliminary categories in each context. This stage revealed strong convergence between the findings, with substantially similar themes in the different institutions. Next, three researchers performed an interinstitutional comparative analysis, which confirmed this thematic consistency. In view of this high similarity and the absence of relevant differences in the findings between the contexts, we chose to present an integrated set of results, as recommended in multicenter qualitative studies in which saturation occurs both within and between the data collection sites.

To ensure methodological rigor in the qualitative analysis, the principles of Reflective Thematic Analysis as proposed by Braun and Clarke (2019, 2021) [19,20] were adopted. Credibility was strengthened by the independent coding carried out by three researchers and by the holding of successive meetings for analytical consensus. Additionally, in a transnational, face-to-face meeting of the HAInnovPrev project, the qualitative analysis of the findings was initially discussed in small groups and then in a plenary session with all those present, involving 27 co-authors of the manuscript, which contributed to expanding the interpretative robustness and consensual validity of the categories developed, as well as the definition of the excerpts from the interviews that represented the category.

Dependability was ensured through the maintenance of a systematic record (audit trail) of analytical decisions, the coding structure and the process of constructing the categories. Confirmability was ensured by continuous reflective discussions among the researchers and by the triangulation of data from the four participating universities. In addition, the analytical team adopted a reflective posture throughout the process, recognizing how their professional and academic experiences could influence the interpretation of the data and actively discussing such influences to mitigate biases and strengthen the coherence of the interpretations. Transferability was contemplated by the detailed description of the simulated scenarios, the institutional context and the characteristics of the participants. These strategies follow the reliability criteria described by Lincoln and Guba [21] and reinforce the robustness and transparency of the qualitative analysis carried out.

### 2.6. Ethical Considerations

The study fully complied with ethical standards for research with human beings and received approval from the institutional ethics committees of the participating universities. Written informed consent was obtained from all participants prior to their involvement. Participants were explicitly informed of their right to refuse participation or withdraw at any point without any academic consequences and were informed that the interviews would be audio-recorded. To protect confidentiality, students received codes that began with the term “Student,” followed by a number and the initials of their respective universities (e.g., Student 1—UFV, Student 2—UFRJ, Student 3—UNACH). The study report complies with the EQUATOR Network guidelines and follows the COREQ checklist for qualitative research [22].

## 3. Results

A total of 480 nursing students participated in the 12 simulated scenarios and the majority was female; however, the focus group interviews were conducted with 297 of these participants (Table 3). Each session lasted approximately 30 to 40 min.

**Figure 1:**
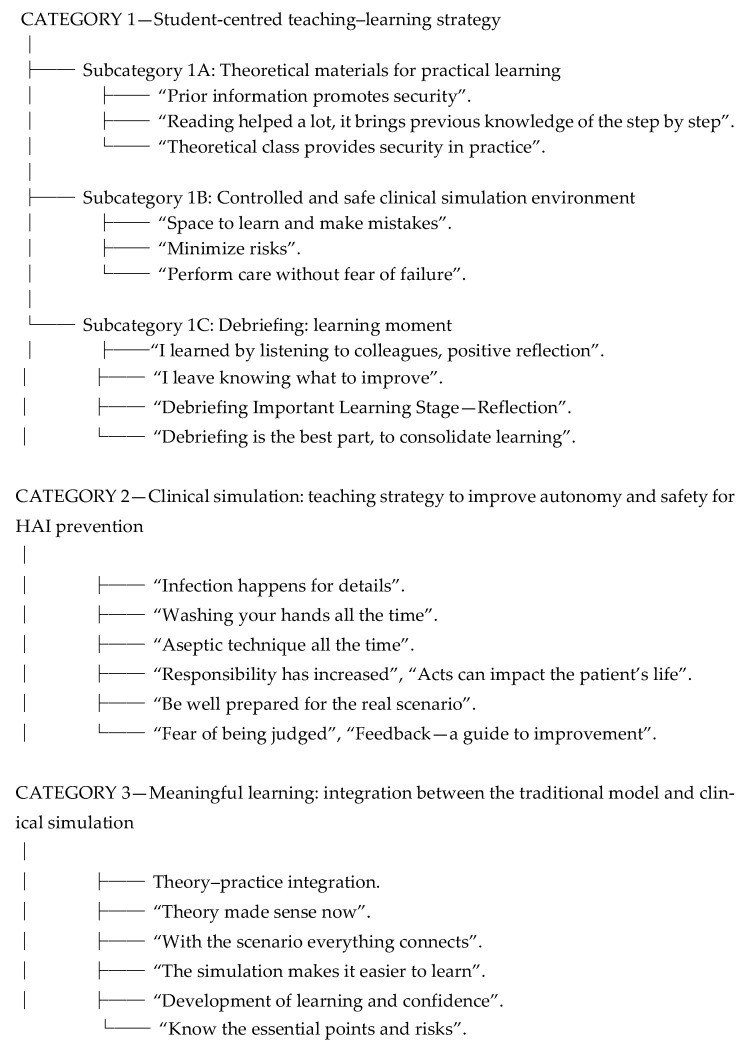
Decision tree of the categories and subcategories emerging from the content analysis of the focus groups. April to October 2024.

Content analysis of the focus group discussions identified three main categories: (i) student-centered teaching–learning strategy; (ii) clinical simulation as a facilitator of autonomy and safety in HAI prevention; and (iii) meaningful learning and integration with the traditional educational model. Each category, along with its subcategories and supporting excerpts from participants’ statements, is described below. Figure 1 presents the decision tree constructed from the content analysis of the focus groups, synthesizing the categories, subcategories and excerpts representative of the participants’ statements.

### 3.1. Category 1: Student-Centered Teaching–Learning Strategy

This category expresses the students’ perception of clinical simulation as a tool that favors an active, safe learning process oriented to the progressive development of competencies. The availability of previous theoretical content, practical experience in a controlled environment and structured debriefing were recognized as essential elements for learning to reduce anxiety and promote critical reflection on performance and safety in care.

#### 3.1.1. Theoretical Materials for Practical Learning

Students valued the theoretical materials provided prior to the practical sessions, recognizing their role in supporting learning. Peruvian students frequently emphasized the need for detailed checklists to ensure no critical steps were omitted, while Brazilian students highlighted how advance reading materials boosted their confidence and reduced anxiety before practice. The following participant statements illustrate this subcategory:

*(…) It would be good to include the steps and all the preventive measures in the process of aspiration of secretions to avoid skipping any important steps and protect the patient’s life.* (Student 2—UNACH)

*The theoretical class and the contents before providing more security for practice. I also believe that if I didn’t have the class, (…) I would have many doubts and divergences of what we remember or not. (…)* (Student 15—UFV)

*Prior distribution of materials also reduces student anxiety and allows for greater comfort. This distribution of the material before the realization of the simulated scenario was great, because it brings prior knowledge of what was going to be done and step by step.* (Student 02—UFRJ)

*Having advanced information allows us to get involved in the development of practice, motivating us to participate in what is planned without doubts or mistakes, (…).* (Student 5—UAC)

#### 3.1.2. Controlled and Safe Clinical Simulation Environment

Participants described the simulation as a safe and controlled environment where they could make mistakes and learn without risk. While Brazilian students stressed the value of practicing without fear of harming patients, Peruvian students noted that the structured environment helped them pay closer attention to procedural details. The following excerpts illustrate this subcategory:

*(…) This is the space for us to really learn, not to make mistakes with the patient”* (Student 08—UFV).

*But what we learn here to execute there in the practice scenario is to promote health, prevent aggravation and minimize risks to the patient, thus, being in a controlled scenario.* (Student 02—UFRJ)

*(…). We can carry out the procedures without fear of failure and with the certainty that they will correct us proactively.* (Student 04—UAC)

*(…) It is of utmost importance in clinical practice and in the development of skills and dexterities to act safely and autonomously in real practice* (Student 4—UNACH)

#### 3.1.3. Debriefing: A Learning Moment

Debriefing was regarded as a crucial moment for consolidating learning. Students in Brazil often emphasized how this stage confirmed their correct practices and built confidence, whereas those in Peru highlighted its role in identifying overlooked steps and reinforcing safety checklists. The following statements illustrate this subcategory:

*The debriefing is one of the most important parts because it allowed the identification of points of improvement, it being a positive and targeted feedback on the activity performed.* (Student 04—UFRJ).

*I think that at the very end (debriefing) was the main thing for us to realize that we did everything right. And I think it was a very cool instrument for us to understand.* (Student 02—UFRJ)

*The debriefing was important to consolidate what we did and review what was done (…)* (Student 07—UFV)

*(…) I consider debriefing to be an important step in the learning process because it allowed us to reflect with tutors and colleagues on what we did well and the aspects that need to be improved. It’s the first time I’ve had this experience, I hope all teachers do it.* (Student 03—UAC)

*(…) the debriefing helped me understand why each step is taken and which part I need to improve* (Student 22—UNACH)

### 3.2. Category 2: Clinical Simulation: Teaching Strategy to Improve Autonomy and Safety for HAI Prevention

Participants emphasized that simulation increased their awareness of risks and attention to detail in nursing care. In Brazil, students frequently reflected on how simulation enhanced their decision-making confidence and reduced fear of errors, while in Peru, students stressed how it revealed the importance of small procedural details and the potential consequences of omissions. The following testimonials illustrate this subcategory:

*(…) Practical and everyday cases help us to better understand the procedures and to be better prepared in the clinical field.* (Student 25—UNACH)

*I think we are also aware of how much a simple act, or even a sequence of acts, can impact a patient’s life.* (Student 05—UFRJ)

*I think that in practice, when we do it, we realize that these infections occur because of details. So, when we see someone doing it, or we do it ourselves, we pay attention and realize how important the details are for the safety of care. (…).* (Student 01—UFV)

*(…) Possible complications should be included, and the nursing professional should manage them during the procedure to be better prepared in a real scenario.* (Student 23—UNACH)

*(…) This controlled environment allowed me to focus on the process, on the application of theoretical knowledge and on learning from our actions; I made mistakes, yes, but the ability to correct and retrospectively analyze them, along with teacher feedback, generated a sense of growth and progress. The feedback, although it sometimes pointed out errors, was not perceived as a negative criticism, but rather as a guide for improvement.* (Student 05—UAC)

### 3.3. Category 3: Meaningful Learning: Integration Between the Traditional Model and Clinical Simulation

Students across both countries agreed that simulation improved comprehension and knowledge retention compared to traditional teaching. Brazilian students frequently described the strategy as motivating and helpful in building self-confidence, while Peruvian students noted how simulation clarified essential steps and reinforced theoretical content through practical application:

*I think that in simulation it is easier to understand and store things than simply the theoretical content. With the simulation, we can know the points that are really essential and the risks, and when it comes to practice, we can remember all the steps taken.”* (Student 04—UFV)

*(…) The simulation scenario was very clear, accurate and motivating, The theoretical material helps us a lot to understand and fix the content to use in practice.* (Student 26—UNACH)

*This is an active strategy, it’s not a passive thing that we just listen to, we just read, we do it (…).* (Student 09—UFRJ)

*(…) I believe that clinical simulation has contributed significantly to the development of learning in students, allowing greater confidence in clinical procedures, as well as safety and improvement of their skills.* (Student 3—UAC).

Across both countries, these perspectives repeated consistently across focus groups, indicating thematic saturation.

## 4. Discussion

This study involved 12 focus groups, with the participation of 297 students, across four institutions in Brazil and Peru, highlighting student protagonism as a fundamental aspect of learning through clinical simulation. Students actively engaged and valued the opportunity to prepare using pre-reading materials. This preparation enhanced their participation in simulated scenarios, increased their confidence and clarity regarding nursing care actions, and improved overall performance. Such anticipation fosters student protagonism and aligns with active learning principles, which emphasize meaningful learning resources and motivation to encourage active involvement, positioning the student at the center of the educational process [23].

Supporting this student-centered approach, the WHO underscores the importance of including simulation in health training curricula, recognizing it as essential for developing skills in HAIs prevention. This recognition further reinforces the value of simulation as a methodological cornerstone in healthcare education [3]. Our findings converge with these recommendations. By strengthening skills in HAIs prevention and patient safety, simulation directly supports the WHO’s call for skill-based curricula and echoes QSEN’s emphasis on safety as a core nursing outcome.

Moreover, evidence from a quasi-experimental study comparing traditional teaching with traditional teaching plus simulation indicates that although both groups showed cognitive improvement, those exposed to simulation achieved significantly greater knowledge gains (*p* = 0.016). This demonstrates the progressive and additive benefits of integrating clinical simulation into traditional curricula [24].

Taken together, these findings suggest that simulation serves as a vital tool to prepare nursing students for the realities of clinical practice. Its benefits extend beyond technical skills, encompassing human interactions with families and healthcare teams, while fostering critical thinking and communication skills essential for safe and effective care [5,8,23]. However, for simulation to be truly effective, careful attention must be given to its design and implementation. This includes deliberate consideration of the clinical scenario, scheduling, scripts, participant roles, materials, and structured pre-briefing and debriefing stages, all of which are crucial for maximizing learning outcomes [15,16,25].

The clinical simulation experience was presented from the student’s perspective, a consolidated learning, and a learning of practical applicability. It can be observed that, as Bandura ponders in his Social Cognitive Theory, there is an emphasis on the active role of people in the construction of their own experiences, with each of the students being an agent of their own development, with active participation in the interaction with the environment, which becomes fundamental for the development of professional competencies and skills [26]. Through the observation of the behavior of other people and the consequences that these actions generate, mediated learning and modeling are verified, which consists of observing the behavior of role models, that is, people who serve as a reference, and the practice of observed behavior [27].

Therefore, simulation appears as a teaching strategy in this educational context, being driven by five elements: clear objectives and prebriefing [14], fidelity and problem solving anchored in the design of the scenario, and support/feedback ensured by structured debriefing [24,25]. The clinical simulation experience, as perceived by the students, was not only a tool for consolidating knowledge but also for applying it in a practical context. This teaching strategy relies on five key elements: clearly defined objectives, effective pre-briefing, scenario fidelity, problem-solving opportunities, and structured, supportive debriefing [15,25,26,27,28]. These components work together to create an engaging and meaningful learning environment.

An important finding was the students’ positive reception of feedback during the debriefing phase. They viewed instructors’ comments as constructive guidance rather than criticism, indicating a psychologically safe learning atmosphere. When well-executed, debriefing promotes critical reflection and strengthens clinical and communication skills. However, some participants expressed concerns about the length and density of the debriefing sessions. This suggests a need to optimize debriefing practices by setting clear objectives, prioritizing key discussion points, managing time effectively, and aligning feedback with critical checklists. Such refinements would preserve the depth of reflection while preventing fatigue and maintaining focus on educational goals [25,26,27,28].

Another noteworthy aspect highlighted by this study is the role of a controlled and safe simulation environment. This space allows students to make mistakes without risking patient harm and to receive formative feedback, which fosters autonomy and clinical reasoning. The fidelity of the simulation setting (i.e., how closely it replicates real clinical conditions) is crucial to this process. Scenarios must be carefully designed with realistic cases and aligned roles for actors and students. Preparation before simulation ensures smooth execution and meaningful interaction [15,25,29]. Importantly, timely feedback after simulation sessions is essential for promoting critical thinking and maximizing the educational value of the experience.

Regarding the development of autonomy and safety awareness, students reported increased attention to detail and risk recognition, both of which are vital to the prevention of HAIs. The simulated environment worked as a strategy for learning about patient safety, where understanding the possible complications reinforced best practices. This highlights the importance of creating realistic scenarios, that is, ones that are in line with institutional, organizational, clinical aspects, based on the best evidence, consider local and international health policies and cultural aspects, so that they meet learning objectives and prepare students for the challenges of the care context [15,25].

The findings of this qualitative study reinforce the effectiveness of the simulation to achieve the proposed objective, by showing greater attention of students to the risks of infection, strengthening confidence to carry out HAI prevention measures, and recognizing the debriefing as a central space for reflection and correction of failures. Similar results were observed by Santos-Costa et al. [14], who demonstrated that the educational approach using simulation for teaching about infection prevention and control favored the consolidation of knowledge and the development of safety attitudes in clinical practice, reinforcing simulation as a relevant teaching strategy for HAI training.

In addition, other evidence also converges with the results of the present study, as it shows that simulation in the teaching of infection prevention and control promoted greater knowledge, improved technical performance and reduced anxiety in students related to care with biological risk [30,31]. In addition, a methodological study on the validation of clinical scenarios for the teaching of HAI shows that the structured design of cases aligned with clear objectives enhances the development of specific competencies in patient safety [32].

Recent evidence indicates that simulation surpasses the results in relation to exclusively expository teaching in the development of skills for infection prevention in health students and stands out among the most effective methodologies for teaching patient safety [5,6,7,8,9,10,11,12,13,14,15,16,17,18,19,20,21,22,23,24,25,26,27,28,29,30,31,32,33]. These findings corroborate the results of the present study, reinforcing the relevance of including simulation in curricula oriented to patient safety and as a central pedagogical resource for nursing education oriented to risk reduction and the strengthening of safe and quality care practices, and aligned with international goals for prevention of data associated with health care.

In the cross-national context of the present study, differences emerged in the way students attributed meaning to the simulation. Peruvian students emphasized the importance of detailed checklists and technical accuracy, reflecting a more structured orientation towards error prevention. Brazilian students, on the other hand, highlighted that the preparation with previous material and the debriefing contributed to increasing confidence and reducing anxiety in the practical application of infection prevention.

According to Rana et al. [34], culture is intrinsically present in the structure of debriefing, shaping the interactions between participants and influencing how feedback is given and received. Since debriefing is a reflective process sustained by collaborative dialogue, it is inevitably permeated by cultural values [35]. Furthermore, cultural differences affect the effectiveness of communication and the ways we teach and learn in simulation environments, which can directly impact openness to dialogue, the interpretation of corrections, and the degree of participation in the reflective process [36]. Recognizing the cultural elements involved in debriefing is essential for achieving the expected educational outcomes and the quality of the training process, especially in multinational teaching scenarios [28].

These findings converge with the literature that shows that cultural norms, communication styles, and institutional practices influence participation and psychological safety during debriefing in simulations, reinforcing the need to pay attention to the context in conducting the activity [34,35,36].

In this sense, when structuring a curriculum that uses simulation as a pedagogical strategy for nursing education, it is necessary to consider local, institutional and organizational aspects, in order to offer structured and reflective support that optimizes educational results [28].

Thus, the use of methods related to constructivist theories is essential. Education that leads to critical development should not be based only on classroom teaching [37]. The teacher should not act as an agent of information transmission, transferring content and depositing it in the students; the interaction with the environment, with people, should occur in order to build knowledge based on previous experiences, like an individual, who, being a knowing subject, unveils a knowable object [38]. In summary, the dialogical, respectful and reflective interaction with teaching professionals generates the possibility of not only building cognitive knowledge, psychomotor skills, but also attitudes, promoting learning and increasing autonomy in the daily exercise of work [38].

Clinical simulation effectively raises students’ awareness of risks and potential errors in patient care, encouraging safer clinical reasoning and decision-making. Its innovative and immersive nature appeals to current generations of learners by combining theory with practice, stimulating critical thinking, and enhancing readiness for real clinical settings [39,40]. Autonomy naturally develops as students are placed in scenarios requiring independent decision-making and reasoning, which are key skills for modern healthcare professionals [29]. The findings support the integration of clinical simulation into nursing curricula focused on HAI prevention. This includes the use of pre-reading materials, goal-oriented briefing, risk-focused scenario execution, and evidence-based, structured debriefing. Such a framework aligns with a growing trend in health education to incorporate simulation, which consistently demonstrates benefits in learners’ confidence, autonomy, and satisfaction [23]. While active participation is central, observation also provides valuable learning opportunities, especially when supported by guided observation tools [41]. This highlights that even students not directly performing procedures can gain meaningful learning, provided observation is structured with clear checklists or reflective prompts.

Patient safety emerged as a central category throughout the participants’ reflections. Simulation nurtures not only technical and cognitive skills but also the attitudes and ethical values necessary for safe, high-quality practice [39]. Evidence supports that simulation-based teaching effectively internalizes safe behaviors (e.g., hand hygiene, proper use of personal protective equipment), and enhances risk recognition, situational awareness, and patient safety competence [5,39]. By providing a protected environment to experiment and reflect on mistakes, simulation fosters a culture of safety. This role has been emphasized by international bodies like the WHO, which advocate for simulation’s inclusion in health professional education to promote patient safety [42,43].

Despite these benefits, traditional lecture-based teaching remains predominant in many nursing programs. Simulation offers a meaningful alternative by actively engaging students in deliberate practice, reflection, and self-evaluation, processes which are often missing from expository methods. Innovative approaches such as “Room of Horrors” scenarios can further enhance learning by encouraging error recognition and confidence building in patient safety [39]. Additionally, emerging technologies like virtual reality simulations provide authentic, engaging experiences that integrate theory and practice while promoting critical thinking [40]. The cross-country perspectives in this study reinforce the need for culturally responsive simulation design. While common benefits were evident, tailoring simulation to local expectations, whether emphasizing confidence-building or procedural detail, may optimize its effectiveness in different institutional and cultural settings.

This study has some limitations to consider. Having only a portion of the simulation participants (297 out of 480) take part in the focus groups may have affected the breadth of perceptions captured. The diversity of institutions across different sociocultural contexts could also influence experiences, which may limit the transferability of findings to other Peruvian, Brazilian, or comparable Latin American settings. The qualitative nature relies on participant spontaneity and researcher facilitation, which can introduce bias. Additionally, as with all focus groups, the possibility of dominant voices influencing group discussions must be acknowledged, which may have shaped the depth or direction of some exchanges. Moreover, not all students took part in every simulated scenario, partly due to time constraints, as noted by the participants. Another limitation was the absence of data triangulation, as the study relied solely on students’ self-reported perceptions, which may have reduced interpretative depth. In addition, it was not possible to compare the profile of the students who participated in the focus groups with those who did not, as well as the reasons for non-participation, which generated selection bias and limited the diversity of perceptions analyzed. Lastly, the feedback about lengthy debriefing sessions suggests the need to optimize this stage for better engagement and depth of reflection. Thus, we believe that future research could adopt quasi-experimental or mixed method designs to quantify simulation’s effects on knowledge, performance, and behavioral outcomes in participants and observers. Testing different debriefing formats and timing may also help maximize learning benefits and reflective depth.

## 5. Conclusions

This multicenter study demonstrated that clinical simulation is a promising strategy for developing skills in the prevention and control of HAIs. The approach fosters psychological safety, provides clear step-by-step guidance supported by pre-reading and briefing, and encourages reflective learning through feedback-driven debriefing. Integrating standardized simulations and structured debriefing across nursing curricula can foster safer clinical practice.

Thus, it is recommended that this incorporation occur longitudinally, that is, throughout the academic training and not just in some disciplines or contents, with simulation activities starting with introductory content, such as hand hygiene and communication, before advancing to care that involves greater technical–scientific complexity, such as airway aspiration and insertion of peripheral venous catheters, and culminating in more complex scenarios, involving decision-making and care mangement. This structure can favor the development and progress of skills in students and prevent simulation from being a methodology to be used in some content or concentrated at the end of the course.

The frequency of the simulated activities may vary according to the curricular design, but it is suggested that they be offered at least once in each module or thematic unit, on a monthly or bimonthly basis, allowing for deliberate practice, review of performance and monitoring of the student’s evolution. In disciplines with greater complexity for patient safety and HAI prevention, for example, simulations are therefore recommended in most contents greater periodicity.

To ensure pedagogical coherence, it is essential to align the simulated scenarios with the competency assessment, considering technical dimensions, such as the correct performance of care; cognitive, involving clinical reasoning and decision-making, for example; and behavioral/attitudinal, such as therapeutic communication, teamwork and risk control. The evaluation may include validated checklists, such as those used in the present study, global performance scales, self-evaluation, peer evaluation, and reflective analysis in the debriefing, allowing continuous monitoring of learning.

In addition, it is essential to establish permanent teacher training programs to ensure mastery of the methodology in each of the stages of the simulation, especially in the briefing, in the writing of the clinical case and conducting the scenario in line with the teaching, social and care context, in the communication of feedback and student-centered debriefing, ensuring consistency and pedagogical quality.

It is suggested that future studies test the longitudinal implementation of simulation in the nursing curriculum in order to measure its effects on competencies, students’ performance in the care context and in different scenarios, as well as outcomes related to patient safety.

## Figures and Tables

**Table 1:** Simulated scenarios implemented by each university. April to October 2024.

University (Country)	Simulated Scenarios
UFV (Brazil) ^1^	1. Prevention of infection transmitted by aerosol and contact. 2. Dressing change on a peripherally inserted central venous catheter (PICC). 3. Prevention and control of urinary tract infections associated with indwelling urinary catheters. 4. Aspiration of secretions in a tracheostomized patient to prevent pneumonia.
UFRJ (Brazil) ^2^	1. Benzathine benzylpenicillin administration in primary health care. 2. Hand hygiene and use of personal protective equipment. 3. Prevention of respiratory tract infections related to care of patients with tracheostomy (peristomal skin cleaning and tracheostomy cannula dressing change). 4. Prevention of peripheral intravenous line infections.
UAC (Peru) ^3^	1. Hand hygiene and use of personal protective equipment. 2. Permanent bladder catheterization. 3. Nursing care for prevention and control of infection transmission in patients under contact isolation. 4. Prevention of peripheral intravenous line infections.
UNACH (Peru) ^4^	1. Aspiration of secretions in a tracheotomy patient to prevent pneumonia. 2. Prevention of simple infections in post-operative wound healing. 3. Preventive education for self-care in safe insulin administration and medical waste management at home. 4. Medication practices in preparation and administration of intravenous drugs.

Note: ^1^ Universidade Federal de Viçosa; ^2^ Federal University of Rio de Janeiro; ^3^ Universidad Andina del Cusco; ^4^ Universidad Nacional Autonoma de Chota.

**Table 2:** Distribution of undergraduate nursing students according to the simulated scenarios and participation in focus groups. April to October 2024.

Simulated Scenarios	Students in the Simulated Scenarios				Total	Students in the Focus Groups				Total
	UFV ^1^ n	UFRJ ^2^ n	UAC ^3^ n	UNACH ^4^ n		UFV n	UFRJ n	UAC n	UNACH n	
Benzathine benzylpenicillin administration in primary healthcare	-	12	-	-	12	-	12	-	-	12
Prevention of peripheral intravenous line infections	-	26	60	-	86	-	26	10	-	36
Prevention of respiratory tract infections associated with the health care of patients with tracheostomy: peristomal skin cleaning and tracheostomy cannula dressing change	-	25	-	-	25	-	25	-	-	25
Aspiration of secretion in a tracheostomized patient to prevent pneumonia.	15	-	-	30	45	15	-	-	30	45
Prevention of infection transmitted by aerosol and contact	17	-	-	-	17	17	-	-	-	17
Change dressing on peripherally inserted central venous catheter (PICC) to prevention of infection associated	17	-	-	-	17	17	-	-	-	17
Hand hygiene and use of personal protective equipment for the prevention and control of healthcare-associated infections	-	12	68	-	80	-	12	10	-	22
Prevention and control of urinary tract infections associated with the use of indwelling urinary catheters	15	-	63	-	78	15	-	10	-	25
Nursing care for the prevention and control of infection transmission in patients in contact isolation	-	-	32	-	32	-	-	10	-	10
Preventive education for self-care in the safe administration of insulin therapy and management of medical waste at home	-	-	-	29	29	-	-	-	29	29
Medication practices in the preparation and administration of intravenous drugs		-	-	30	30	-	-	-	30	30
Prevention of simple postoperative wound infections	-	-	-	29	29	-	-	-	29	29
Total	64	75	223	118	480	64	75	40	118	297

Note: ^1^ Universidade Federal de Viçosa; ^2^ Federal University of Rio de Janeiro; ^3^ Universidad Andina del Cusco; ^4^ Universidad Nacional Autonoma de Chota.

**Table 3:** Sociodemographic characteristics of nursing students participating in the simulated scenarios. (n = 480). April to October 2024.

Sociodemographic Characteristics		Total Sample (n = 480)	UFV ^1^ (n = 64)	UFRJ ^2^ (n = 75)	UAC ^3^ (n = 223)	UNACH ^4^ (n = 118)
Age (years) (média + dP)		21.8 + 3.5	22.5 + 3.5	23.9 + 4.2	21.4 + 3.5	20.6 + 1.8
Gender	Femele	400	53	66	197	84
	Male	80	11	09	26	34
Course Semester (Students)	First	49	17	12	-	20
	Second	83	-	12	45	26
	Third	98	-	51	27	20
	Fourth	88	-	-	56	32
	Fifth	76	32	-	24	20
	Sixth	40	-	-	40	-
	Seventh	33	15	-	18	-
	Eighth	13	-	-	13	-

Note: ^1^ Universidade Federal de Viçosa; ^2^ Federal University of Rio de Janeiro; ^3^ Universidad Andina del Cusco; ^4^ Universidad Nacional Autonoma de Chota.

## Data Availability

The original contributions presented in this study are included in the article. Further inquiries can be directed to the corresponding author.

## Public Involvement Statement

No public involvement in any aspect of this research.

## Guidelines and Standards Statement

This manuscript was drafted against the Consolidated Criteria for Reporting Qualitative Research (COREQ): A 32-item checklist for interviews and focus groups for qualitative research [22]. A complete list of reporting guidelines can be accessed via the equator network: https://www.equator-network.org/ (accessed on 3 January 2026).

## Abbreviations

The following abbreviations are used in this manuscript:

CBHE	Capacity Building in the field of Higher Education
COREQ	Consolidated criteria for Reporting Qualitative research
EACEA	European Union or the European Education and Culture Executive Agency
Erasmus+	European Union cooperation programme for education
HAInnovPrev	Name of the Erasmus+ project cited in the study
HAIs	Healthcare-associated Infections
WHO	World Health Organization
PICC	Peripherally Inserted Central Catheter
QSEN	Quality and Safety Education for Nurses
UAC	Universidad Andina del Cusco (Peru)
UFRJ	Federal University of Rio de Janeiro (Brazil)
UFV	Federal University of Viçosa (Brazil)
UNACH	Universidad Nacional Autónoma de Chota (Peru)
